# BASE: a practical *de novo* assembler for large genomes using long NGS reads

**DOI:** 10.1186/s12864-016-2829-5

**Published:** 2016-08-31

**Authors:** Binghang Liu, Chi-Man Liu, Dinghua Li, Yingrui Li, Hing-Fung Ting, Siu-Ming Yiu, Ruibang Luo, Tak-Wah Lam

**Affiliations:** Bioinformatics Algorithms Research Laboratory, Department of Computer Science, University of Hong Kong, Pokfulam, Hong Kong

## Abstract

**Background:**

*De novo* genome assembly using NGS data remains a computation-intensive task especially for large genomes. In practice, efficiency is often a primary concern and favors using a more efficient assembler like SOAPdenovo2. Yet SOAPdenovo2, based on *de Bruijn* graph, fails to take full advantage of longer NGS reads (say, 150 bp to 250 bp from Illumina HiSeq and MiSeq). Assemblers that are based on string graphs (e.g., SGA), though less popular and also very slow, are more favorable for longer reads.

**Methods:**

This paper shows a new *de novo* assembler called BASE. It enhances the classic seed-extension approach by indexing the reads efficiently to generate adaptive seeds that have high probability to appear uniquely in the genome. Such seeds form the basis for BASE to build extension trees and then to use reverse validation to remove the branches based on read coverage and paired-end information, resulting in high-quality consensus sequences of reads sharing the seeds. Such consensus sequences are then extended to contigs.

**Results:**

Experiments on two bacteria and four human datasets shows the advantage of BASE in both contig quality and speed in dealing with longer reads. In the experiment on bacteria, two datasets with read length of 100 bp and 250 bp were used.. Especially for the 250 bp dataset, BASE gives much better quality than SOAPdenovo2 and SGA and is simlilar to SPAdes. Regarding speed, BASE is consistently a few times faster than SPAdes and SGA, but still slower than SOAPdenovo2. BASE and Soapdenov2 are further compared using human datasets with read length 100 bp, 150 bp and 250 bp. BASE shows a higher N50 for all datasets, while the improvement becomes more significant when read length reaches 250 bp. Besides, BASE is more-meory efficent than SOAPdenovo2 when sequencing data with error rate.

**Conclusions:**

BASE is a practically efficient tool for constructing contig, with significant improvement in quality for long NGS reads. It is relatively easy to extend BASE to include scaffolding.

## Background

The past few years have witnessed a number of improved *de novo* genome assemblers, providing users choices between speed and accuracy [1]. The more recent NGS technologies have gradually increase the read length beyond 100 bp (e.g., 150 bp from HiSeq and 250 - 400 bp from MiSeq), yet existing efficient assemblers do not have much improvement regarding accuracy, and it remains challenge how to take better advantage of longer NGS reads to assemble genomes in a fast and accurate manner. This paper presents a new assembler that can achieve better assembly quality for longer reads when compared with those efficient assemblers, without scarifying speed a lot.

Most state-of-the-art short read assemblers such as SOAPdenovo2 [2] and ALLPATHS-LG [3] are based on *de Bruijn* graph (DBG). In these assemblers, reads are chopped into a sequence of overlapping k-mers such that two adjacent *k*-mers have *k*-1 bases in common. The DBG based method works well for short reads but it is non-trivial to handle repetitive sequences that are longer than *k*. When the reads are longer, it is natural to consider using a larger *k*, yet this is not feasible as this will require higher sequencing depth and consume much more memory (especially for NGS data with high error rate). Another method is to use the multiple *k*-mer strategies like IDBA-UD [4] and SPAdes [5], which could save memory by using smaller *k* parameter to take care most of sequencing errors, and using larger *k* to solve longer repetitive sequences. Yet this requires multiple constructions of DBG and much longer running time, limiting their usage for the assembly for relative large genome.

Assemblers for Sanger sequencing reads or Roche 454 reads (400-800 bp) are mostly based on Overlap-Layout-Consensus (OLC) strategy, such as Celera assembler and Newbler. An alternative representation named string graph was proposed by Mayer a decade ago [6], which has been implemented in assemblers such as SGA [7], Fermi [8], and Readjoiner [9]. Like OLC based assemblers, a proper minimum overlap size is required in string graph based assemblers to reduce the complexity of graph and to improve the connectivity of graph. Smaller minimum overlap size will increase the probability that the overlap sequence falls within a repetitive region of the genome. This leaves much more branches in the graph and may result in shorter contigs. Meanwhile, according to the Lander-Waterman model [10], larger minimum overlap size leads to a reduction of sufficient support for overlap among reads, thus enhancing the demand for higher sequencing depth. Therefore, due to the variation in length of repetitive sequences in genome, it is difficult to find a fixed minimum overlap size that fits all use cases especially when the NGS read is not so long. Regarding speed, for 30X 100 bp reads, BASE took 2 days and 5 days to assemble raw contigs by SGA [7] and Fermi [8], respectively. This speed is much slower than DBG based assembler SOAPdenovo2, which takes only a half day to obtain raw contigs.

**Fig. 1:**
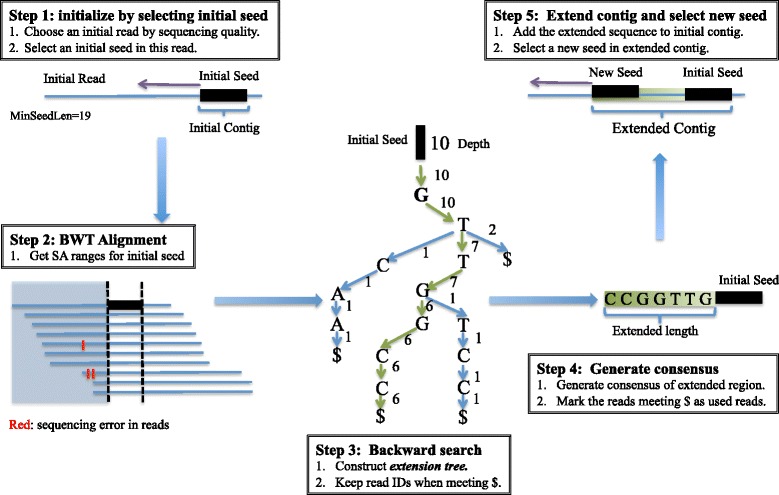
Overview of the whole assembly method. There are five steps for one direction extension. Firstly, we choose an initial read by order and find an initial seed in this read. Then we use bi-directional BWT to get the SA ranges of this seed using backward exact matching. Thirdly, we build up a backward extension tree by adding bases to continue the backward matching. After removing erroneous branches and heterozygosis branches, we obtain the consensus sequence of the extended region. Finally, we continue to find a new seed in the extended region and extend iteratively

Recently, a very efficient GPU-based method has been developed to index short reads using the Burrows Wheeler Transform (BWT) or bi-directional BWT of short reads [11]. For 30X human short reads, it only needs 6 h to build the BWT index. With such an index, the depth of any sequence that is no longer than the read length could be calculated in real time, which enables us to predict whether a sequence comes from a repetitive region of the genome [12]. With such efficient indexing, we find that it becomes feasible to produce better assemblies efficiently for large genomes using longer NGS reads, and in particular, we developed an adaptive seed extension method called BASE to construct contigs by searching for non-repetitive overlaps between reads. The details of our algorithm are given in the Methods Section, and an overview of the extension method is shown in Fig. 1. We tested the performance of BASE using data from HiSeq and MiSeq, with length ranging from 100 bp to 250 bp and compared BASE to popular assemblers including SOAPdenovo2, SGA and SPAdes.

## Methods

### Preliminary

Given a set of reads *R* = {*R*_*0*_*, R*_*1*_*, …, R*_*n-1*_}, and each *R*_*i*_ is terminated with a sentinel symbol $ (i.e., *R*_*i*_[|*R*_*i*_|] = $). We also define *R*_*i*_[|*R*_*i*_|] < *R*_*j*_[|*R*_*j*_|], if *i* < *j*.

Let Suff_*R*_ = {*Ri*[*j*…|*R*_*i*_|] | 0 ≤ *i* < *n* and 0 ≤ *j* ≤ |*R*_*i*_|} be all possible suffices of reads in *R*. The suffix array SA_*R*_ of *R* is defined as SA_*R*_[*k*] = (*i*, *j*) if *R*_*i*_[*j*…|*R*_*i*_|] is the *k*-th lexicographical smallest suffix in Suff_*R*_. The BWT of *R* is an array defined by BWT_*R*_[*k*] = *R*_*i*_[*j*-1] if SA_*R*_[*k*] = (*i*, *j*).

Given a string *P*, the range [l_*R*_(*P*), u_*R*_(*P*)] of *P* in SA_*R*_ is defined as follows:

- l_*R*_(*P*) = min{*k* | SA_*R*_[*k*] = (*i*, *j*) and *P* is a prefix of *R*_*i*_[*j*…|*R*_*i*_|]}
- u_*R*_(*P*) = max{*k* | SA_*R*_[*k*] = (*i*, *j*) and *P* is a prefix of *R*_*i*_[*j*…|*R*_*i*_|]}

From this definition, the size of SA range (u_*R*_(*P*) -l_*R*_(*P*) + 1) is the number of reads containing string *P*. If l_*R*_(*P*) > u_*R*_(*P*), it means that *P* is not a substring of any reads in *R*.

For double-stranded DNA sequence, we define *P'* as the reverse sequence of *P* and RC(*P*) for its reverse complement sequence. In this way, we also define *R’* as the reverse of *R*, SA_*R’*_ as the suffix array of *R’* and BWT of *R’*. Then for string *P*, we can also have the SA' range as [l_*R'*_(*P'*), u_*R'*_(*P'*)], which can help to find the reads containing the RC(*P*) [13]. The BWT of *R* and BWT of *R’* form the bi-directional BWT of *R.*

For bi-directional BWT, we introduce the term *intact SA range* (ISR) of *P*, which is the combination of: a) the SA range of *P* in *R*, b) the SA range of RC(*P*) in *R*, and c) the SA' range of RC(*P*) in *R*. The *intact SA range* is denoted by ISR(*P*) = [l_*R*_(*P*), u_*R*_(*P*), l_*R*_(RC(*P*)), u_*R*_(RC(*P*)), l_*R'*_(RC(*P*)), u_*R'*_(RC(*P*))]. Note that the size of b) and c) are the same. With respect to ISR(*P*), the depth of *P* (with respect to the set *R*) is defined as follows:

- Dep(*P*) = max{0, u_*R*_(*P*) - l_*R*_(*P*) + 1} + max{0, u_*R'*_(RC(*P*)) - l_*R'*_(RC(*P*)) + 1}.

As shown by Lam et al. [13], bi-directional BWT can finish the following operations in constant time:

- For any string *P* and a character *c* in {A, C, G, T, $}, calculate the SA range of *cP* from the SA range of *P.*
- For any string *P* and a character *c* in {A, C, G, T, $}, calculate the SA range and the SA' range of *cP* (or *Pc*) from the SA range and the SA' range of *P.*

Then for *P* = *P*[0…*m*], using backward searching, the depth of *P*[*m*], *P*[*m*-1…*m*],…, *P*[0…*m*] can be calculated incrementally by updating their ISRs. Therefore, with the bi-directional BWT of *R*, it is possible to trace how Dep(*P*) decreases with the increasing length of *P*.

### Bi-directional BWT construction

We build the BWT index of the reads based on our GPU-accerlated algorithm [11]. To test the efficiency of GPU acceleration and to make this construction available to computers without GPU, we have also developed a CPU-only version.

Meanwhile, the detailed content of BWT is also modified for easier genome assembly.

- A base is encoded with 4 bits using the first 3 bits to encode “A”, “C”, “G”, “T” or the read terminal symbol “$”, and the last 1 bit to indicate whether the base has a high sequencing quality with respect to a user-defined threshold.
- Read ID (2*i*, 2*i* + 1) is defined by the *i*-th pair of reads, and an auxiliary table is used to record the mapping between a read ID and the position of the “$” in the BWT w.r.t this read. This enables fast recovery of read sequences and qualities in linear time. However, this method requires that all reads have equal length.

### Seed selection

A seed is a sub-sequence shorter than a read. The main idea of our seed selection strategy is to select the seeds that have only one occurrence in the genome to be assembled. In the context of *de novo* assembly, there is no way to calculate the exact number of occurrences of a seed in the genome. We develop the following method to guarantee a high probability to select one-occurrence seeds, which we call *inferred-unique* seeds.

Let *d* be the average sequencing depth of a genome, and each read has length *m*. Here we define the expected depth of a sub-sequence *P* with length *k* to be *d*_*k*_ = (m – k + 1) * d/m [12]. If Dep(*P*) (which is the depth of *P* calculated according to ISR(*P*) in Section 2) is no larger than z*d_*k*_, in which *z* is a user-defined parameter, we define *P* as an *inferred-unique* sequence, which means it is likely to occur only once in the genome.

To find an *inferred-unique* seed in a read *R*_*i*_ or a previously extended sequence, starting at the end and by using backward search mentioned above, we can update the ISRs and calculate the depth incrementally until it achieves *inferred-unique*. For example, we find a seed in read *R*_*i*_ of length *m,* we calcualte the ISRs and depth of *R*_*i*_*[m-1], R*_*i*_*[m-2,m-1], …, R*_*i*_*[1…m-1]* and *R*_*i*_*[0…m-1].* Meanwhile, We calculate the expected depth *d*_*m-j*_ with *j* decreasing from *m-1* to *0*. Then there would be two cases for the changes of depth from these sub-sequences:

Case 1: The depth of *R*_*i*_[*j*…*m-1*] is reduced to less than user-defined depth threshold *τ*. Then we will further try to find seed in the substring *R*_*i*_[*0*…*j-1*].

Case 2: The depth of *R*_*i*_[*j*…*m-1*] is no larger than z* d_*m-j*_*.* Then sub-sequence *R*_*i*_[*j*…*k-1*] meets the requirement of *inferred-unique* and no more sub-sequences will be checked.

Each *inferred-unique* sub-sequence will be further checked to make sure the all the bases in seed have high quality scores (using the 1-bit base quality stored in BWT). Then we obtain a high quality *inferred-unique* seed to start the extension step. It can be found in a read to initialize an extention as an *initial seed* or in the extended regions to start a new iteration.

### Seed extension and consensus

Given a pattern *P* with Dep(*P*) > 0 and a character *c*, we define *cP* as a valid backward extension of *P* if and only if Dep(*cP*) > 0. For a seed *S*, by adding characters in the head base by base, we can construct a backward extension tree *T*_*s*_ whose nodes are tagged with characters chosen from {“A”, “C”, “G”, “T”}, except for the root node, which is tagged with Seed *S*. Define *L(v)*, the label of a node *v,* to be the concatenation of tags from *v* to the root; and define *W*(*v*), the weight of the node *v,* to be the depth of *L*(*v)*.

Backward extension tree is built recursively. The root tagged with *S* is firstly created. For each newly created node *v*, if *cL(v)* is a valid backward extension of *L(v)* for some character *c* in {“A”, “C”, “G”, “T”}, a new node is created as a child of *v* and is tagged with *c*. Note that the label of a node will not be longer than the read length, the depth of the tree is limited by the read length minus the seed length. Moreover, for any node *v* in the tree, if Dep($*L(v)*) > 0, we obtain the IDs of reads which have *L(v)* as a shared prefix and mark these reads to avoid redundant assembly.

**Fig. 2:**
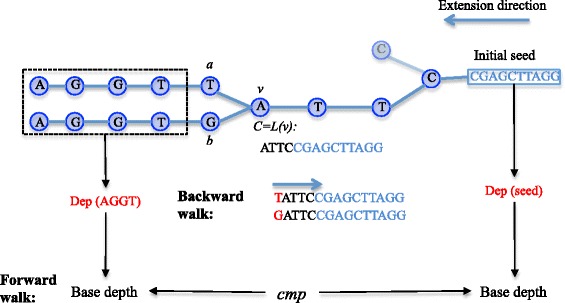
Remove branches in backward extension tree. In the backward extension tree, we try to remove erroneous branches, repetitive branches and heterozygosis branches to obtain the consensus sequences of the extended region. As an example, we meet node *v* with two child node *a* and *b*. Firstly, combined with *L*(*v*), we obtained T*L*(*v*) for *a* and G*L*(*v*) for *b* to detect erroneous branches between *a* and *b*. We incrementally calculate the depth of sub-sequences of *a*(sub-*a*_*i*_ with length *i*): T, TA, TAT, …, and b(*sub*-*b*_*i*_ with length *i*): G, GA, GAT, … until the depth of sub-*a* is less than user-defined threshold *τ*. At the same time, if Dep(sub-*a*_*i*_) is significantly smaller than Dep(sub-*a*_*i-1*_), Dep(sub-*a*_*i*_) is significantly smaller than d_i_ and Dep(sub-*b*_*i*_) is significantly larger than Dep(sub-*a*_*i*_), then branch *a* will be treated as a erroneous branch or repetitive branch. When there is no erroneous signal, we will further try to remove the branch, which might be caused by heterozygosis. After obtaining two sequences representing the consensus sequences of the sub-trees rooted at *a* and *b* respectively, we compare the two sequences to find the matched region and get the depth of it. Then we use this depth to calculate base depth and compare to the base depth calculated by depth of *initial seed*. If the two sequences have high similarity and the two base depths are similar to each other, we will treat *a* as heterozygous branch if *W*(*a*) is smaller than *W*(*b*)

The consensus sequence for the backward extension tree is constructed by walking down the tree from the root to a certain node. This process is called *consensus-walk*. When visiting a node with only one child, the walk moves on to that child. Otherwise we have to select a branch to move on or stop immediately. A greedy algorithm, which chooses the child with the largest weight, is straightforward but error-prone. Therefore, we introduce another strategy, which we call *reverse validation,* to improve the probability of choosing the correct branch. For simplicity, we describe our method for the case of two branches. As shown in Fig. 2, let *ν* be the node that the consensus is currently processing to, *a* and *b* be two children of *v*, tagged with *t*_*a*_ and *t*_*b*_ respectively. Let *C* = *L*(*v*) be the consensus sequence we have already constructed. The method incrementally calculates the depth of *t*_*a*_, *t*_*a*_*C*[0], *t*_*a*_*C*[0 … 1], *t*_*a*_*C*[0 … 2], etc. and *t*_*b*_, *t*_*b*_*C*[0], *t*_*b*_*C*[0 … 1], *t*_*b*_*C*[0 … 2], etc.

Below *τ* denotes a user-defined threshold.

Case 1. If Dep(*t*_*a*_*C*[0 … *i*]) < *τ* and Dep(*t*_*a*_*C*[0 … *i*]) < 0 for some *i*, we immediately conclude that *a* is an erroneous branch and *b* is authentic if it demonstrates the following properties:

- Dep(*t*_*a*_*C*[0 … *i -* 1]) is significantly larger than Dep(*t*_*a*_*C*[0 … *i*]).
- Dep(*t*_*b*_*C*[0 … *i*]) is significantly larger than Dep(*t*_*a*_*C*[0 … *i*]).
- The expected depth *d*_*i+2*_ is significantly larger than Dep(*t*_*a*_*C*[0 … *i*]).

Case 2. if Dep(*t*_*a*_*C*[0 … *i*]) = 0 for some *i*, we conclude that the initial seed is a false positive *inferred-unique* seed and *a* is near another copy of this seed in the genome, and *a* is named as a repetitive branch if it demonstrates the following properties:

- Dep(*t*_*a*_*C*[0 … *i -* 1]) is significantly larger than 0.
- Dep(*t*_*b*_*C*[0 … *i*]) is significantly larger than 0.
- *d*_*i*+2_ is significantly larger than 0.

If we fail to identify the above two cases, an additional step will be introduced to estimate whether the branches are due to heterozygous sites. Starting from *a* and *b*, we use a greedy algorithm mentioned above to obtain two sequences representing the consensus of the sub-trees rooted at *a* and *b,* respectively. If the similarity of these two sequences is high enough, we make a prediction that these two branches are caused by heterozygous sites, and walk to the child with larger weight. Otherwise, the *consensus-walk* stopped at node *v*.

If the consensus-walk does not stop at the root of the tree, i.e. the consensus sequence has been extent by at least one base pair from the seed, a new *inferred unique* seed will be chosen from the prefixes of the consensus sequences to start a new round. The process of seeding, backward extension and consensus is repeated until the *consensus-walk* stops at the root of the extension tree in some round. Then a series of symmetric processes follow, which forward extend the *initial seed*. Finally the contig containing this *initial seed*, which is the concatenation of the consensus sequences in both directions, is obtained when the forward extension completes.

### Contig assembly using paired-end information

Paired-end reads have adjacent read IDs in the BWT and this information is used to resolve longer repetitive regions, when the *consensus-walk* stops at the root of the extension tree. For two children nodes *a* and *b* of root node *v*, reads with “$” falling in the sub-tree of *a* and sub-tree of *b* regions are divided into *R(a)* and *R(b)*. We check whether the paired reads of *R(a)* or *R(b)* have been used in current assembled sequences and whether the distances are proper as estimated by their positions in this contig and their insert sizes. Without loss of generality, if only paired reads of *R(a)* are found and the number is larger than user-defined threshold *τ* mentioned above, the child node *b* will be removed. This method could be used to assemble repetitive sequences longer than read length and obtain longer contig sequences.

## Results and discussion

### Datasets

We compared the assembly performance based on several sets of real data, including two bacterial *Staphylococcus aureusMW2* 240X HiSeq 100 bp reads (SRR857914) [14], *V. parahaemolyticus* 240X MiSeq 250 bp reads (DRX016227) [15], and four human sequencing data sets including YH Solexa 100 bp reads [2] (gigadb.org), YH HiSeq 150 bp reads (BGI), NA12878D HiSeq X Ten 150 bp data (DNAnexus.com) and NA12878 HiSeq 250 bp data (SRR891258, SRR891259). All raw sequencing data are pre-processed with SOAPfilter [2] to remove reads containing excessive amount of ‘N’s or adaptors, low quality reads and duplicated reads. The four human datasets are further corrected with SOAPec [2] using 23-mer.

### Evaluation

Using reference genomes for *Staphylococcus aureus* MW2 (www.genomic.ch/edena/results2013/ReferenceSequences/) and *V. parahaemolyticus* (RIMD2210633), we evaluated the accuracy of assembly using the GAGE pipeline [16], in which metrics such as correct N50, mismatch, align rate and coverage are assessed. For YH and NA12878, we mapped the assembled contigs to hg19 with LAST [17] and evaluated the alignment rate, reference coverage and repeat-masked reference coverage.

### Comparison

As shown in the bacterial assembly (Table 1), BASE obtains contigs with the highest accuracy among all evaluated assemblers and is the only assembler that achieve 100 % alignment rate. Except four translocations of SPAdes in dataset of *V.para,* translocations assembled by BASE, SGA and SOAPdenovo2 are all caused by circular DNA and are not included in Table 1. For the 100 bp dataset of *S.aureus*, the correct N50 statistics of BASE is much shorter than that of SPAdes and is only a bit longer than that of SGA and SOAPdenovo2. Further analysis showed that BASE’s improvement over SGA and SOAPdenenovo2 is mainly due to the effective usage of paired-end information. For the 250 bp dataset of *V.para*, the correct N50 from BASE is indeed comparable to that of SPAdes and is much longer than that of SGA and SOAPdenovo2. As shown in Table 1, BASE takes slightly longer time in building index and assembling contigs than SOAPdenovo2, but is much faster than SPAdes and SGA. The coverage of contigs from BASE is relatively low, which could be improved by devoting more time to initialize more extension or by scaffolding like SOAPdenovo2.

**Table 1:** Contig assembly of deeply sequenced bacterial genomes

	Tools	Parameters	Correct N50	Misatch/Indel	Aligned rate	Coverage	Time(sec)
*S.aureus* MW2 (240X, 100 bp HiSeq)	SPAdes	51,63,85	299,305	134/6	99.79 %	100.00 %	1239
	SOAPdenovo2	87-95	82,495	0/0	99.84 %	99.27 %	25;16
	SGA	29;91	74,584	7/0	99.81 %	99.98 %	1228;1149
	BASE	4	92,706	0/0	100.00 %	99.97 %	161; 93
*V.para* (240X, 250 bp MiSeq)	SPAdes	33,55,65,75,85,99	169,978	118/45	99.97 %	99.97 %	4616
	SOAPdenovo2	125	88,858	23/30	99.98 %	99.98 %	110;1
	SGA	29;149	95,711	58/26	99.80 %	99.97 %	2478;2884
	BASE	4	159,715	29/29	100.00 %	99.75 %	676; 388

*S.aureus* MW2 has its real reference with length 2.8 Mb and *V.para* has its species’ reference with length 5.1 Mb and two chromosomes. Both of these two bacteria are sequenced up to 240X. GAGE validation pipeline was used to calculate the corrected contig N50, base errors, structural errors, contig aligned rate and reference coverage. Except BASE used single thread for contig assembly part, and other the assemblies were all performed with 24 threads. The time before semicolon is for index building and after semicolon is for assembly. For SGA, indexing time contains the time used in the indexing after error correction and filtering; assembly time contains the time used in the overlap and assembly

**Fig. 3:**
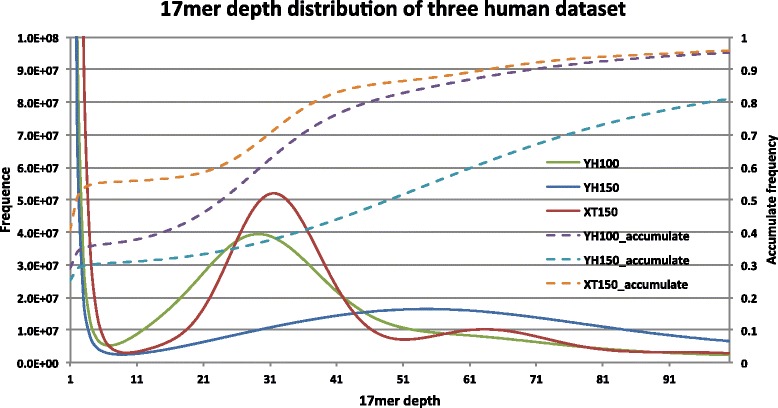
17mer depth distribution of three human sequencing dataset. We count the depth of all 17mers in the sequencing reads, and calculate the frequency of each depth. About 35 % 17mers of YH 100 bp reads, 30 % 17mers of YH 150 bp reads and 53 % 17mers of NA12878D XTen reads having depth no more than 3. Then we say the NA12878D XTen reads should have more sequencing errors left after raw data filter and correction than YH dataset

We also tested human genome assembly with four datasets: YH 100 bp ~35X, YH 150 bp ~63X, NA12878 XTen 150 bp ~35X and NA12878 HiSeq 250 bp ~45X. With 30X 100 bp reads, it already took SGA more than 2 days [7] and Fermi nearly five days [8] to output the contigs. As shown in Table 2, for the ~35X 100 bp YH dataset, both BASE and SOAPdenovo2 (in single-kmer mode) took only about half a day to obtain the contigs. To assemble X Ten data (150 bp reads), BASE used much less memory than SOAPdenovo2 on indexing and contig assembly (Table 3). This is probably related to the high error rate of X Ten data, as shown in 17mer depth distribution of the three datasets in Fig. 3.

**Table 2:** Performance for human genome assembly

		SOAPdenovo2			BASE		
		Wall time (h)	CPU time (h)	Max memory (GB)	Wall time (h)	CPU time (h)	Max memory (GB)
YH, 100 bp, 36X	Index	4	46	163	5	18	200
	Contig	2	2	41	4	53	140
	Total	6	48	163	9	71	200
YH, 150 bp, 64X	Index	6	75	201	11	36	192
	Contig	1	1	33	5	80	225
	Total	7	76	201	16	116	225
NA12878D, 150 bp	Index	9	141	477	9	34	194
	Contig	1	1	24	7	144	142
	Total	10	141	477	16	178	194

For X Ten data, we used a different machine with larger memory to finish SOAPdenovo2 and BASE assembly, so it is improper to compare the time usage of this dataset to other dataset. Other dataset are all performed in the same machine with 24 CPU

**Table 3:** Summary of human contig assembly

	YH, 100 bp		YH, 150 bp		NA12878, 150 bp		NA12878, 250 bp	
	SOAPdenovo2,k = 41	BASE	SOAPdenovo2,k = 61	BASE	SOAPdenovo2,k = 41	BASE	SOAPdenovo2,k = 61	BASE
Contig num	3,420,897	3,319,617	2,279,026	2,145,792	8,068,278	1,934,261	1,416,658	1,511,270
Contig size	2.67E + 09	2.88E + 09	2.76E + 09	2.95E + 09	2.44E + 09	2.90E + 09	2.60E + 09	2.94E + 09
Contig N50	2,244	2,279	3,008	3,126	1,140	3,823	3,368	4,199
Contig aligned rate	99.10 %	97.07 %	98.87 %	95.96 %	99.40 %	97.62 %	99.34 %	96.33 %
Genome coverage	90.36 %	93.76 %	93.12 %	93.90 %	84.11 %	95.58 %	89.55 %	94.09 %
RepeatMasked coverage	97.05 %	96.13 %	97.28 %	95.32 %	93.94 %	97.38 %	95.60 %	95.99 %
Exon coverage	93.76 %	91.51 %	95.73 %	94.13 %	91.48 %	96.84 %	93.90 %	91.49 %
Mismatch base	2,735,141	3,479,046	2,911,990	3,839,110	2,301,111	3,459,648	2,544,785	3,751,887
Mismatch ratio	0.103 %	0.121 %	0.105 %	0.130 %	0.094 %	0.119 %	0.098 %	0.128 %
Indel num	340,930	327,469	358,358	334,989	259,190	322,214	327,695	372,941
Indel base	1,412,005	1,587,265	1,692,213	1,741,947	1,086,014	1,602,240	1,400,230	1,953,311
Indel ratio	0.053 %	0.057 %	0.062 %	0.061 %	0.045 %	0.057 %	0.054 %	0.069 %

We mapped the raw contigs to Hg19. Aligned rate is the contig-aligned length divided by total contig length. To calculate genome coverage, the length of gap regions in Hg19 has been removed. For unique coverage, the repetitive regions have been further removed. For SOAPdenovo2 contig assembly, we all used single-kmer method and M1 to treat heterozygous regions

In all four human datasets, shown in Table 3 the N50 statistics of BASE improves as read length increases, while SOAPdenovo2 does not show such degree of improvement. BASE’s improvement over SOAPdenovo2 becomes significant for 250 bp reads. Similar to bacterial assembly, BASE’s genome coverage, with repeat masked, is lower that of SOAPdenovo2. But BASE has an overall higher genome coverage in each dataset. This suggests that BASE is able to assemble more repetitive regions, which can be used to explain the slightly more mismatches between assembled sequences and hg19 for BASE, as shown in Table 4.

**Table 4:** Mismatches analysis for human genome assembly

Dataset	Assembler	Whole genome mismatches				Exon region mismatches			
		Total	Variant Call	Public SNP	Novel	Total	Variant Call	Public SNP	Novel
YH_100bp	SOAPdenovo2	2,735,141	2,423,482	108,044	203,615	47,926	40,701	1,590	5,635
	BASE	3,479,046	2,872,396	208,351	398,299	47,515	42,124	1,724	3,667
YH_150bp	SOAPdenovo2	2,911,990	2,613,670	113,037	185,283	46,002	43,151	1,148	1,703
	BASE	3,839,110	3,075,913	256,217	506,980	52,561	46,420	1,822	4,319
NA12878_150bp	SOAPdenovo2	2,301,111	2,025,220	109,497	166,394	39,702	35,644	1,740	2,318
	BASE	3,459,648	3,052,269	129,933	277,446	49,711	45,361	1,151	3,199
NA12878_250bp	SOAPdenovo2	2,544,785	2,122,144	130,806	291,835	42,744	35,890	1,936	4,918
	BASE	3,751,887	2,613,065	604,805	534,017	48,635	37,853	5,068	5,714

We mapped the assembled contigs to Hg19 and got the mismatches between each contig and reference sequence. Then we used the detected SNPs and SNPs from published SNP databases to analysis these mismatches in whole genome and exon regions respecitively

The efficiency of GPU acceleration is shown in Table 5. To construct BWT of small datasets from two bacterial genomes, without GPU, it takes 1.38 ~ 1.56 folds longer time than GPU version. But for large human datasets, the CPU-only version takes near 4 times as much time as the GPU version. Therefore the GPU version is recommended especially for large sequencing dataset.

**Table 5:** Acceleration performance of GPU on BWT construction

	Read_num	Read_length	With GPU	Without GPU	Time fold
*S.aureus MW2*	6,720,000	100	133 s	184 s	1.38
*V.para*	4,896,000	250	329 s	514 s	1.56
YH, 100 bp	1,057,750,382	100	5 h	19 h	3.80
NA12878, 150 bp	770,960,980	150	7 h	30 h	4.29

To evaluate the acceleration performance of GPU on BWT construction, we used two versions, one of which used GPU and the other not, to build bi-directional BWT on four datasets. The results showed that especially in large genome dataset, compared with GPU version, version without GPU costs ~4 fold more time to construct BWT

## Conclusions

The primary objective of this paper is to study whether a seed-extension approach to contig assembly, coupled with reverse validation, can give a performance (accuracy and N50) comparable to SOAPdenovo2 and SGA. As shown in the previous section, the new approach gives clear advantage for longer reads, and with speed much higher than SGA and comparable to SOAPdenovo2, and stable memory usage (i.e., not sensitive to error rate of the reads). The contigs obtained by BASE are longer and cover more repetitive sequences than those from SOAPdenovo2 and SGA.

Based on the high quality contigs assembled by BASE, one could use less accurate third generation reads or paired-end reads with longer insert size for scaffolding and gap closing. This approach has been used in a recently published assembler DBG2OLC [18], which assembles second level contigs onto high accurate DBG contigs. Indels or SV could also be detected with these contigs using established methods [8].

With the increasing length of high quality Illumina reads, it is of computational interest how to fully utlilize the read length information in contig assembly. SGA, Fermi and our tool BASE both build an index of the reads and make it possible to assemble high-depth short reads without splitting them into kmers. Although SGA and Fermi could finish assembly with less memory, they need much longer time. As noted in MEGAHIT [19], the requirment for big memory machine can be circumvented. For future bioinformatics analysis including assembly, it is time and robustness that matter most. We plan to further reduce the running time of BASE.
